# Agitation and apathy increase risk of dementia in psychiatric inpatients with late-onset psychiatric symptoms

**DOI:** 10.1186/s12888-021-03210-5

**Published:** 2021-04-28

**Authors:** Yuan Shao, Haiyun Xu, Jian Wang, Xijian Dai, Wei Liang, Lina Ren, Yongjun Wang

**Affiliations:** 1 Shenzhen Mental Health Center, Shenzhen Kangning Hospital, Shenzhen, China; 2 The Affiliated Kangning Hospital of Wenzhou Medical University, Wenzhou Medical University, Wenzhou, China

**Keywords:** Agitation, Apathy, Dementia, Neuropsychiatric symptoms, Non-demented psychiatric disorders

## Abstract

**Background:**

A diagnosis of dementia in middle-aged and elder people is often complicated by physical frailty and comorbid neuropsychiatric symptoms (NPSs). Previous studies have identified NPSs as a risk factor for dementia. The aim of this study was to figure out to what extent individual NPS and certain demographic factors increased the risk of dementia in middle-aged and senior psychiatric inpatients.

**Methods:**

One hundred twenty-seven middle-aged and senior patients admitted to psychiatric wards for late-onset (age ≥ 50 years) psychiatric symptoms were included and categorized into dementia or non-demented psychiatric disorders (NDPD). The patients’ demographic information and medical records were collected during the first hospitalization and subjected to statistical analyses.

**Results:**

41.73% of the registered psychiatric inpatients were diagnosed as dementia in which Alzheimer’s disease (AD) was the dominant subtype. The NDPD group consisted of nine individual diagnoses, except for schizophrenia. The frequencies of dementia inpatients increased with first episode age while that of NDPD inpatients decreased with first episode age. In the enrolled inpatients, most of dementia patients were males while females accounted for a higher proportion of NDPD patients. 58.49% of enrolled dementia inpatients presented cognitive deficit (CD) as the initial symptom while the remaining 41.51% showed NPS as initial symptom. Of the 12 NPSs, agitation and apathy greatly and significantly increased risk of dementia in psychiatric inpatients with late-onset psychiatric symptoms.

**Conclusions:**

These results added evidence that the demented patients admitted to psychiatric ward are more likely to be male, older first episode age, and have characteristic NPS including aberrant motor behavior (AMB), hallucinations, agitation, irritability and apathy. Further, this study emphasized the importance of agitation and apathy of NPSs functioning as risk factors of dementia in these inpatients.

**Supplementary Information:**

The online version contains supplementary material available at 10.1186/s12888-021-03210-5.

## Background

Dementia is a common neurodegenerative disorder characterized by progressive cognitive impairment and decreased ability of daily living [1], with or without behavioral and psychological symptoms of dementia (BPSD).

Neuropsychiatric symptoms (NPSs) have been viewed as “non-cognitive” symptoms of dementia, encompassing impairments of mood, anxiety, drive, perception, sleep, appetite, and behavioral disturbances such as agitation or aggression [2]. Previous studies have shown associations between NPSs and poorer outcomes in dementia patients manifesting as greater caregiver burden [3], higher rates of institutionalization, poorer quality of life [4], and accelerated progression to severe dementia or death [5]. On the other hand, NPSs were identified as a risk factor for dementia [6]. Specifically, the presence of NPSs increased the incidence of dementia in patients with mild cognitive impairment (MCI), a prodrome of AD [7]. Previous studies exhibited that people who presents anxiety, negative affect, hostility, pessimism, hopelessness, and perceived constraints were at a 20–30% increased risk of dementia [8]. Anxiety has been previously associated with both cognitive decline [9] and risk of dementia [10]. All these findings support the point that NPSs may be viewed as risk factors for dementia.

Relevant to MCI, mild behavioral impairment (MBI) was proposed as MCI of the frontotemporal type [11]. MBI is an umbrella concept describing a syndrome in which late-onset psychiatric symptoms, including major depression, generalized anxiety, delusional disorder and so forth are early manifestations of neurodegenerative disease. The updated MBI criteria describes that MBI is a syndrome of BPSD starting later in life (age ≥ 50 years) and persisting at least intermittently for 6 months [12]. The details of the criteria consist of (1) persistent behavioral changes and mild psychiatric symptoms, especially disinhibition, (2) no serious cognitive complaints, (3) normal activities of daily living, and (4) absence of dementia [13–15]. More importantly, MBI was hypothesized to increase the risk for dementia, especially for FTLD, whether or not significant cognitive symptoms are present. Indeed, Taragano and colleagues proved that over 70% of patients with MBI developed into dementia [16]. These studies suggest that clinicians should pay close attention to the psychiatric patients started with NPSs over 50 years old.

Despite of the advances in MBI and NPSs mentioned above, no one assessed the association between each NPS and dementia, especially in middle aged and senior patients with late-onset psychiatric symptoms. The present study aimed to figure out to what extent individual NPS and certain demographic factors increased the risk of dementia in psychiatric inpatients with first episode age over 50 years. These patients were diagnosed as various types of dementia or non-demented psychiatric disorders (NDPD) when they were discharged from the psychiatry department. The two groups of patients were compared on demographic information and medical records to explore associations between these variables and dementia and NDPD in the patients investigated. Furthermore, we figured out the risks of some demographic factors and certain NPS for dementia using binary logistic regression analysis.

## Methods

### Participants

Participants of this study were 127 middle-aged and senior patients admitted to psychiatric wards of Shenzhen Kangning Hospital for late-onset (age ≥ 50 years) psychiatric symptoms during June 3, 2019 to April 30, 2020. All patients underwent a standard screening including history, Mini-mental State Examination Scale (MMSE), assessment of daily living activities, and assessment of NPSs. The demographic information and medical records of the patients were collected. The medical records included the results of above mentioned assessments, patients’ initial symptoms, discharge diagnosis, and comorbidities. All data were obtained at the first hospitalization. The initial symptoms included NPSs and cognitive symptoms. Comorbidities included diabetes, dyslipidemia, hypertension, and myocardial infarction. The Shenzhen Kangning Hospital Ethics Committee approved the study design, data collection, and publication of the results.

The discharge diagnosis of patients was done according to the International Classification of Diseases, 10th Revision (ICD-10). The patients were diagnosed as dementia (AD, DLB, FTLD, Parkinson’s disease dementia, VD, dementia due to neurosyphilis, or MCI) or NDPD (acute and transient psychotic disorder, bipolar disorder, delirium, delusional disorder, depression, mental disorder due to alcohol use, somatization disorder, stress-related disorder, or the other mental disorders due to brain damage and physical diseases). The various types of dementia were diagnosed on the basis of established and accepted medical criteria for AD [1], DLB [17], FTLD [18], VD [19], and MCI [20].

### Assessment of cognitive function

The assessments of cognitive function done for all the patients include clinical feature assessment and the neuropsychological scale assessment. The former encompassed complex attention, executive function, learning and memory, language, as well as perceptual-motor and social cognition. Patients with functional impairment in two or more domains were thought to be cognitively impaired. The neuropsychological scale assessment was performed by operating the Chinese version of MMSE. According to the Chinese nationwide norms of MMSE [21], the cut-off point in this study was set at 24. It was important to note that MMSE assessment was only used as a reference basis, not the diagnostic criteria, for dementia diagnosis.

### Assessment of daily living activities

The assessment of daily living activities was based on the Chinese version of Activities of Daily Living Scale (ADLs) [22]. ADLs consists of 6 general activity of daily living (toileting, feeding, dressing, grooming, locomotion and bathing) and 8 instrumental activity of daily living (ability to use telephone, shopping, food preparing, housekeeping, laundry, mode of transportation, responsibility for own medication, and ability to handle finance). Each of the 14 items was rated in terms of severity (ranging from 1 to 4). The total score of ADLs ranges from 14 to 56 points and higher ADLs scores indicate lower activities of daily living. An ADLs score more than 22 points was defined as having impairment in activities of daily living.

### Assessment of NPSs

The assessment of NPSs was performed using the neuropsychiatric inventory questionnaire (NPI-Q). The NPI-Q was answered by the caregiver at the time of admission. It consists of a retrospective (up to 1 month) assessment of 12 neuropsychiatric symptoms commonly present in dementia [23, 24]: including delusions, hallucinations, agitation/aggression, depression/dysphoria, anxiety, elation/ euphoria, apathy/indifference, disinhibition, irritability/lability, aberrant motor behavior, sleep and nighttime behavior disorders, as well as appetite and eating disorders. Each of the 12 symptoms was rated in terms of severity (ranging from 0, absent, to 3, severe). The total NPI score ranges from 0 to 36 and higher scores indicate more severe psychopathology.

### Data analysis

Data analysis was done using the SPSS version 17.0 (SPSS Inc., Chicago, IL) with *P* < 0.05 as the significant level. The *Kolmogorov-Smirnov* test was used to test the normality of all variables. Student’s t-test was done to compare group differences of continuous variables (mean ± SD) while the Chi square test for categorical variables (%). Binary logistic regression was performed to figure out the risk of individual demographic factor and certain NPS with dementia in enrolled psychiatric inpatients. And binary logistic regression analysis was done with the adjustment for age and sex to calculate OR values of the demographic factor and NPSs.

## Results

### Demographic and clinical data of enrolled dementia and NDPD patients

**Fig. 1:**
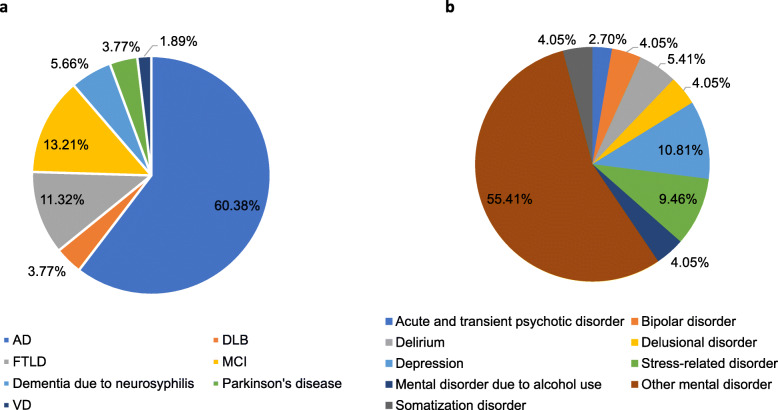
Frequency distribution of dementia subtypes (**a**) and individual NDPD (**b**)

Of the 127 patients with late-onset (age ≥ 50 years) psychiatric symptoms, 53 (41.73%) and 74 (58.27%) people were diagnosed with dementia and NDPD, respectively. The 53 dementia patients consisted of 32 AD cases, 7 MCI, 6 FTLD, 3 dementia due to neurosyphilis, 2 DLB, 2 Parkinson’s disease, and 1 VD. The frequency distribution of these dementia subtypes is shown in Fig. 1a., while the frequency distribution of NDPD is shown in Fig. 1b.

**Fig. 2:**
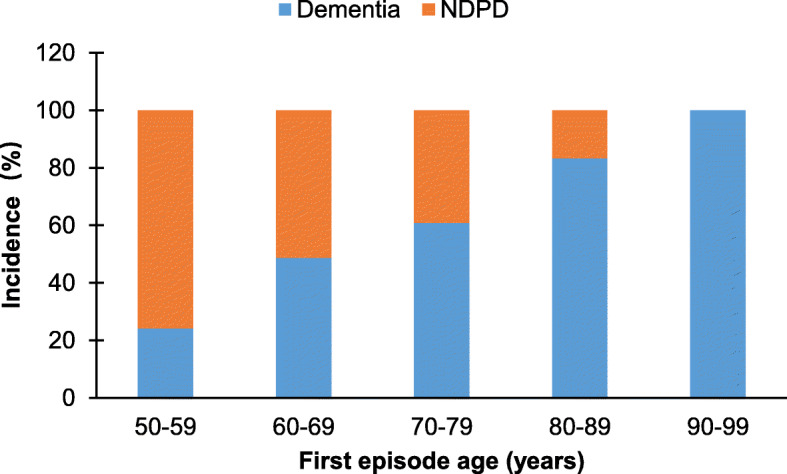
The incidences of dementia and NDPD in each age group

All the 127 patients were in a wide first episode age range over 50 years old. We checked the frequencies of dementia and NDPD inpatients and calculated the incidences of the two major diagnoses in each age group. As shown in Fig. 2, the incidence of participated dementia patients increased with first episode age, whereas NDPD patients decreased with first episode age. In addition, comorbidities in dementia and NDPD inpatients were diagnosed and the incidence of each comorbidity in dementia and NDPD inpatients was compared. As shown in Supplementary data, the two groups were comparable in terms of the incidences of all the comorbidities including diabetes, dyslipidemia, hypertension, and myocardial infarction.

The incidences of dementia and NDPD inpatients in each age group indicate the presence of two opposite but age-related morbidities of these two major diagnoses in middle-age and elder psychiatric inpatients. To further analyze this age-related difference, we set a first episode 65-years-old cutoff. In addition, we compared the two major diagnoses in terms of the other important variables including sex, family history, initial symptom, as well as scores of ADL and MMSE. As shown in Table 1, most (52.83%) of first episode dementia inpatients were elder than 65-years, while majority (81.08%) of NDPD inpatients were less than 65 years old. Of the dementia group, males accounted for a higher proportion (54.72%) than females did (45.28%). In contrast, most of NDPD inpatients were females (64.86%) admitted for the other mental disorders due to brain damage and physical diseases (56.25%), while depression (14.58%) and stress-related disorder (6.25%) accounted for relatively small portions (data not shown). Dementia inpatients presented cognitive deficit (CD) or NPSs as initial symptom, though the former case happened at a relatively higher incidence (58.49%) than the latter case did (41.51%). In comparison, the vast majority (94.59%) of NDPD inpatients presented NPSs as initial symptom.

**Table 1:** Demographic and clinical data of enrolled dementia and NDPD patients

Variables	Dementia patients (***n*** = 53)	NDPD patients (***n*** = 74)	t/x^**2**^	***p value***
1st episode, N (%)				
≤ 65 years	25 (47.17)	60 (81.08)		
≥ 66 years	28 (52.83)	14 (18.92)	16.05^b^	<0.001
Sex, N (%)				
Male	29 (54.72)	26 (35.14)		
Female	24 (45.28)	48 (64.86)	4.82^b^	0.028
Comorbidities, N (%)				
Yes	25 (47.17)	36 (48.65)		
No	28 (52.83)	38 (51.35)	0.03^b^	0.869
Family history, N (%)				
Positive	14 (26.42)	16 (21.62)		
Negtive	39 (73.58)	58 (78.38)	0.39^b^	0.531
Initial symptom, N (%)				
Cognitive deficit	31 (58.49)	4 (5.41)		
NPSs	22 (41.51)	70 (94.59)	43.59^b^	<0.001
MMSE scores (mean ± SD)	14.85 ± 9.38	26.12 ± 8.90	6.82^a^	<0.001
ADL scores (mean ± SD)	29.09 ± 9.12	17.28 ± 8.22	7.63^a^	<0.001

^a^means Students t-test, ^b^ means Chi square test

Dementia inpatients presented a higher averaged ADL score (29.09 ± 9.12) compared to the prior established cutoff (22 points), indicating the patients had ADL impairment. These patients also suffered cognitive impairment as evidenced by an averaged MMSE score of 14.85 ± 9.34, which is lower than the cutoff point of 24. In contrast, NDPD inpatients showed a higher averaged MMSE score (26.12 ± 8.90), indicating that the patients appeared to be normal in the neuropsychological scale assessment. These patients were normal in ADL assessment as their ADL score (17.28 ± 8.22) was lower than the cutoff point of 22.

### Risk factors for dementia in psychiatric inpatients with late-onset psychiatric symptoms

In addition to demographic and clinical data, we compared the incidence of each NPS in dementia inpatients and those with NDPD. As shown in Table 2, the two major diagnoses were different in the incidences of certain NPS. Specifically, the incidences of aberrant motor behavior (AMB), agitation, apathy, hallucination, and irritability in dementia inpatients were significantly higher than the corresponding comparator in NDPD group, while the others were comparable between the two groups. Consequently, dementia group had a significantly higher NPI score (10.34 ± 4.48) than the NDPD group (8.58 ± 3.40). These data suggest that these NPSs may increase the risk of dementia in middle-aged and elderly inpatients with late-onset psychiatric symptoms.

**Table 2:** Neuropsychiatric symptoms in enrolled dementia and NDPD patients

NPI	Dementia patients (n = 53)	NDPD patients (n = 74)	x^**2**^	***p value***
Aberrant motor behavior	36 (67.92)	32 (43.24)	7.56	0.006
Agitation	30 (56.60)	21 (28.38)	10.24	0.001
Anxiety	13 (24.53)	20 (27.03)	0.10	0.752
Apathy	30 (56.60)	13 (17.57)	21.01	<0.001
Appetite	13 (24.53)	30 (40.54)	3.54	0.060
Depression	13 (24.53)	23 (31.08)	0.65	0.419
Delusion	27 (50.94)	36 (48.65)	0.07	0.799
Disinhibition	20 (37.74)	21 (28.38)	1.24	0.266
Elation	5 (9.43)	6 (8.11)	0.07	0.793
Hallucination	18 (33.96)	11 (14.86)	6.39	0.011
Irritability	40 (75.47)	41 (55.41)	5.38	0.020
Nighttime behavior	37 (69.81)	56 (75.68)	0.54	0.462
NPI score	10.34 ± 4.84	8.58 ± 3.40	4.84	0.026

Data were expressed as n (%)

The above data analysis results indicate that first episode age, sex, initial symptom, as well as AMB, agitation, apathy, hallucination, and irritability were significantly associated with the incidence of dementia in the middle-aged and senior inpatients in this study. Our next job was to confirm these associations and specify to what extent each of these factors increased the risk of dementia in psychiatric inpatient with late-onset psychiatric symptoms. For this purpose, binary logistic regression was performed with these variables. As shown in Table 3, all the factors significantly increased the risk of dementia in the psychiatric inpatients. Of them, initial symptom had the highest OR value of 24.66 (95% CI = 7.84–77.58) followed by age with an OR of 7.49 (95% CI = 3.25–17.26). Of the NPSs, apathy showed the highest OR value of 6.12 (95% CI = 2.73–13.74). After adjusted for gender and age, the effect of irritability became insignificant, the effects of AMB (*p* = 0.072) and hallucination (*p* = 0.061) became marginal, only agitation and apathy remained to be high risk factors of dementia in psychiatric inpatient with late-onset psychiatric symptoms.

**Table 3:** Risk factors for dementia in psychiatric inpatients with late-onset psychiatric symptoms

Variables	Unadjusted		Adjusted for age and sex	
	OR (95% Cl)	***p*** value	OR (95% CI)	***p*** value
Age (years) (≥66 vs ≤65)	7.49 (3.25–17.26)	<0.001	9.09 (3.71–22.26)	<0.001
Sex (M vs F)	2.23 (1.08–4.59)	0.029	3.14 (1.34–7.37)	0.009
Initial symptom (CD vs NPS)	24.66 (7.84–77.58)	<0.001	57.42 (10.54–312.88)	<0.001
Hallucination	2.95 (1.25–6.94)	0.013	2.62 (0.96–7.19)	0.061
Agitation	3.29 (1.57–6.91)	0.002	3.49 (1.45–8.39)	0.005
Apathy	6.12 (2.73–13.74)	<0.001	6.97(2.62–18.51)	<0.001
Irritability	2.48 (1.14–5.38)	0.022	1.98 (0.83–4.75)	0.124
AMB	2.78 (1.33–5.81)	0.007	2.16 (0.93–5.01)	0.072

*AMB* aberrant motor behavior, *CD* cognitive deficit, *F* female, *M* male, *NPS* neuropsychiatric symptom

## Discussion

In the present study, 41.73% of psychiatric inpatients with late-onset psychiatric symptoms were diagnosed with dementia, indicating a high incidence of dementia among these patients. This result is in line with previous studies reporting that 40% of psychoses in elder adults were due to AD and other dementias [25]. The subtypes of dementia found in the psychiatric inpatients included AD (60.38%), DLB (3.77%), FTLD (11.32%), MCI (13.21%), dementia due to neurosyphilis (5.66%), Parkinson’s disease (3.77%), and VD (1.89%). These figures are different from the population-based studies on the prevalence of dementia showing that VD was the second most common cause of dementia followed by FTLD [26, 27]. Certainly, this difference could be attributed to the fact that we selected psychiatric inpatients with late-onset psychiatric symptoms in present study. This is a highly selected group, not necessarily representative of demented patients as a whole. The frequencies of different dementias simply reflect their admissions in this study. So the subtype of dementia patients may or may not be overrepresented in a psychiatric inpatient population.

The NDPD in this study consisted of nine individual diagnoses, except for schizophrenia, indicating a very low prevalence of schizophrenia in psychiatric inpatients with first episode age over 50 years old. This interpretation is consistent with previous studies showing that the incidence of first episode schizophrenia was 0.03% in people over 40 [28]. In another research, the prevalence of very late-onset schizophrenia was only 0.1–0.5%, and the patients were characterized by psychotic symptoms such as delusions and hallucinations [29]. In contrast, people over 50 years old showed a high incidence of dementia. Previous studies suggested that FTLD most occured at 45–65 years old [21]. Women over 65 years old have a 7% probability of suffering from AD. And for every 5 years of age increases, the risk of dementia doubles [22]. MBI was also at a high risk (up to 71.5%) of developing dementia [23]. These findings suggest that dementia had a high incidence in the psychiatric inpatients with first-episode mental disorder after 50 years old.

As expected, the incidence of dementia patients increased with first episode age in the present study. This finding is in accordance with a recent age-related prevalence estimates for dementia in the UK, reporting a prevalence of 1.3% in the entire UK population, but 7.1% in those aged 65 or over [30]. In addition, we found that males accounted for a higher proportion relative to females in dementia patients. This seems to be different from the same population-based study in UK showing that men and women under 85 developed dementia at comparable prevalence, and females had a higher prevalence than males after 85 years old. This inconsistence is not surprising as the UK study is a population-based study which had a large number of older persons over 85. The present study is a clinical research with a relatively small sample having only a small number of patients over 85 years of age and therefore did not represent the entire population of China. However, our finding that most (64.86%) of NDPD inpatients were females is in line with the previous studies pointing out the association of female sex, among the other factors, with some psychiatric disorders (including depression, and stress-related disorder) in older adults [31, 32].

It is not surprising that 58.49% of dementia patients presented CD as initial symptom while the remaining 41.51% showed NPS as initial symptom. These results are meaningful and will benefit the early diagnosis of dementia, not only by emphasizing the importance of assessment of cognitive functions for suspected patients, but also reminding family members and caregivers of NPSs that may present in advance of CD. Indeed, the diagnosis of dementia in older adults can be challenging to primary care providers because early symptoms of dementia like memory impairment may not be apparent and minor forgetfulness may be difficult to be distinguished from normal ageing. In contrast, the vast majority (94.6%) of NDPD inpatients presented NPSs as initial symptoms, confirming the usefulness of NPI in identifying this major diagnosis. In support of the accurateness of the two major diagnoses, patients with NDPD presented an averaged MMSE score (26.12), which is slightly higher than the cutoff point (24), whereas they had an ADL score of 17.28 which is lower than the established cutoff indicating that the NDPD inpatients had no mild impairment in daily living activities.

The main aims of this study were to figure out the association of each NPS with dementia and specify to what extent individual NPS increased the risk of dementia in psychiatric inpatients started with NPSs over 50 years old. For the first aim, we compared the total score of NPI and incidence of each symptom in dementia inpatients to those with NDPD. We found that dementia group had a significantly higher NPI (10.34 ± 4.48) than patients with NDPD, confirming that mild NPSs is a common phenomenon in enrolled dementia inpatients, even more common/severe compared to NDPD inpatients. These results added evidence that NPSs are exceedingly prevalent in dementia patients hospitalized in psychiatric ward [33, 34]. Indeed, noncognitive symptoms of dementia occur in 98% of individuals at some point during their disease process as reported in clinical settings [35]. Furthermore, we found that the incidences of individual NPS were different between the two major diagnoses. Specifically, AMB, agitation, apathy, hallucination, and irritability presented at higher frequencies in dementia inpatients relative to those with NDPD, whereas appetite and eating disorder happened in NDPD inpatients more frequently. The two groups were comparable in incidences of the remaining NPSs.

The results of binary logistic regression revealed that agitation and apathy remained to be high risk factors of dementia in late-onset psychiatric inpatient after adjusted for gender and first episode age. This concluding result is in line with a previous population-based study which evaluated the frequency of symptoms in people with dementia and reported that apathy was the most frequent symptom, followed by depression and agitation [36]. The majority of patients in the cited study was AD, along with VD, Parkinson’s disease dementia, and others. These are coincidently similar to the dementia subtypes in the present study. This coincidence reminds us to be cautious when generalizing the conclusion, i.e., the presentation frequencies of each individual NPS in dementia patients may vary depending on patient population. In support of this reminding, a previous study with patients admitted to a geriatric psychiatry unit in Germany reported that aggression was the most frequent symptom (seen in approximately 57% of patients) [37]. In the other studies, however, apathy was the most common (affecting up to 76% of patients) and lasting NPS in AD patients [36, 38].

While recognizing the clinical relevance of the results shown here, we were aware of the limitations of this study. First, the sample of this study was relatively small thus did not allow further analysis on each subtype of dementia and individual NDPD. Second, for the same reason, it did not allow powerful statistical analysis on the comorbidity data which showed no inter-group differences. Third, the diagnoses lacked solid supports from sophisticated laboratory and radiological tests, some of which are very expensive in China and must be paid out of patients’ pocket. Forth, MMSE is a cursory test to assess the cognitive impairment in different domains. It has obvious “ceiling effect” and “floor effect” leading to low specificity and sensitivity. The memory item of it is not enough to be considered a validated assessment of the memory domain. Nonetheless, the data presented in this study encourage us to further investigate the diagnosis and treatment of patients with dementia and NDPD in future studies, in which cognitive function evaluation of the patients will be done by more reliable and specific methods.

## Conclusions

To sum up, 41.73% of psychiatric inpatients with late-onset (age ≥ 50 years) psychiatric symptoms were diagnosed with dementia in which AD was the dominant subtype. The NDPD consisted of nine individual diagnoses, except for schizophrenia. The incidence of dementia inpatients increased with first episode age, whereas that of NDPD decreased with first episode age. Most of dementia inpatients were males while females accounted for a higher proportion in NDPD. 58.49% of dementia inpatients presented CD as initial symptom while the remaining 41.51% showed NPS as initial symptom. Of the 12 NPSs, agitation and apathy greatly and significantly increased risk of dementia in psychiatric inpatients with late-onset psychiatric symptoms.

## Additional file

**Additional file 1: Supplementary data.** The incidences of comorbidities in enrolled patients with dementia or NDPD.

## Data Availability

The data that support the findings of this study are not publicly available due to the sensitive nature of the information. Anonymous summary data can be made available from corresponding author upon request. Restrictions apply to sharing the sensitive individual-level human data which are regulated by the approvals from Ethics and Data protection agency limiting the use of the data. Individual-level data can be shared if the necessary Ethics and Data protection approvals are obtained.

## Abbreviations

AD: Alzheimer’s disease
ADLs: Activities of daily living scale
AMB: Aberrant motor behavior
BPSD: Behavioral and psychological symptoms of dementia
CD: Cognitive deficit
DLB: Dementia with lewy bodies
FTLD: Frontotemporal lobar dementia
MBI: Mild behavioral impairment
MCI: Mild cognitive impairment
MMSE: Mini-mental state examination scale
NDPD: Non-demented psychiatric disorder
NPI-Q: Neuropsychiatric inventory questionnaire
NPS: Neuropsychiatric symptom
VD: Vascular dementia
