# Supplementary figures and images for: Agitation and apathy increase risk of dementia in psychiatric inpatients with late-onset psychiatric symptoms
Source: BMC Psychiatry. 2021 Apr 28;21:214. doi: 10.1186/s12888-021-03210-5 (PMC8080316; doi:10.1186/s12888-021-03210-5)

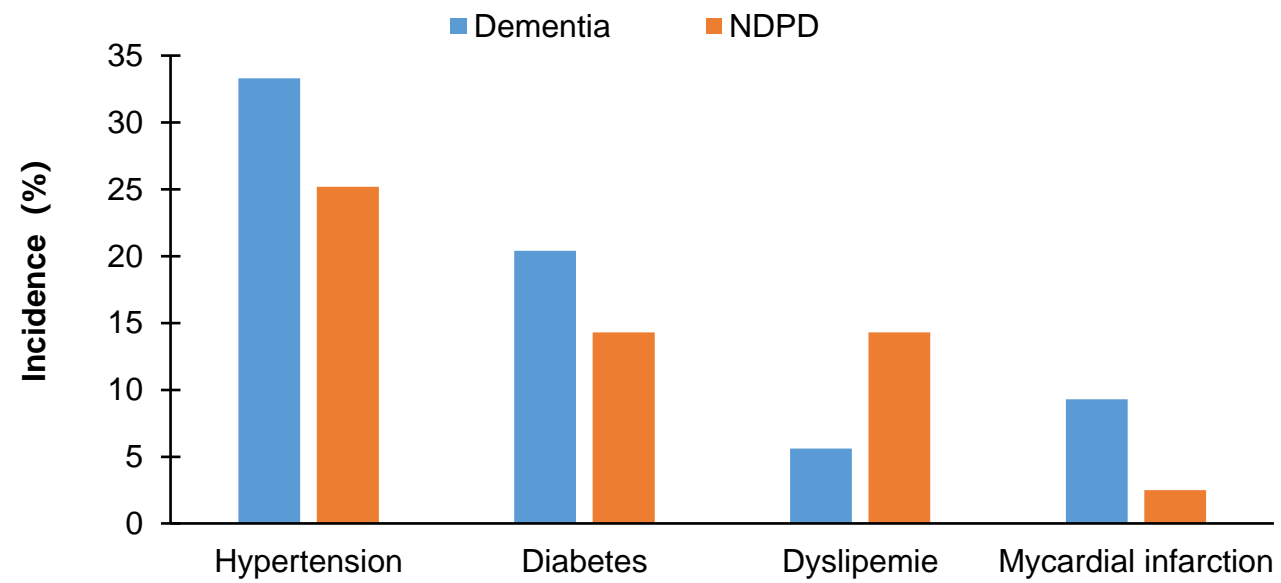

Supplement: Supplementary file 1 — Additional file 1: Supplementary data. The incidences of comorbidities in enrolled patients with dementia or NDPD. [file 12888_2021_3210_MOESM1_ESM.pdf]
